# Challenging retrieval of a large gastric adenocarcinoma after endoscopic submucosal dissection

**DOI:** 10.1055/a-2738-7141

**Published:** 2025-11-21

**Authors:** Kara Ingram, Ameya Deshmukh, Marina Kim

**Affiliations:** 1 12274 Saint Louis University School of Medicine, Saint Louis, Missouri, United States; 2 25213 Division of Gastroenterology and Hepatology, Saint Louis University Hospital, Saint Louis, Missouri, United States

Endoscopic submucosal dissection (ESD) allows en bloc resection of early-stage gastric adenocarcinoma. However, the removal of large lesions poses technical challenges, particularly with passage through the lower esophageal sphincter (LES) and the upper esophageal sphincter (UES). Manipulation in these areas can provoke sphincter spasm, increasing the risk of specimen fragmentation or mucosal injury. This case describes successful en bloc resection and retrieval of a large gastric adenocarcinoma originating from the lesser curvature of the gastric body, using an ESD approach to safely navigate LES-related challenges ( Video 1 ).

Demonstration of en bloc retrieval of a 40 mm gastric adenocarcinoma following ESD, using a double-channel scope and retrieval hood to safely navigate the LES.Video 1

A 54-year-old woman was found to have a 40 mm adenocarcinoma (Paris ISP, JNET 2B) on the lesser curvature of the proximal gastric body ( Fig. 1 ). The lesion had a thick 4 cm stalk, and neoplastic tissue extended close to the base of the stalk (within 15 mm), making ESD the preferred method for resection. Lesion margins were demarcated using high-definition white light, narrow band imaging, and thermal marking. The submucosal lift was achieved with a premixed solution of methylene blue, saline, and epinephrine. Circumferential incision and submucosal dissection were performed with a 2.0 mm electrosurgical knife. Dissection was complicated by submucosal fibrosis and multiple large-caliber vessels, which were treated with large 5 mm coagulation graspers. The 40 mm lesion was resected en bloc, and the defect was closed with eight hemostatic clips.

**Fig. 1:**
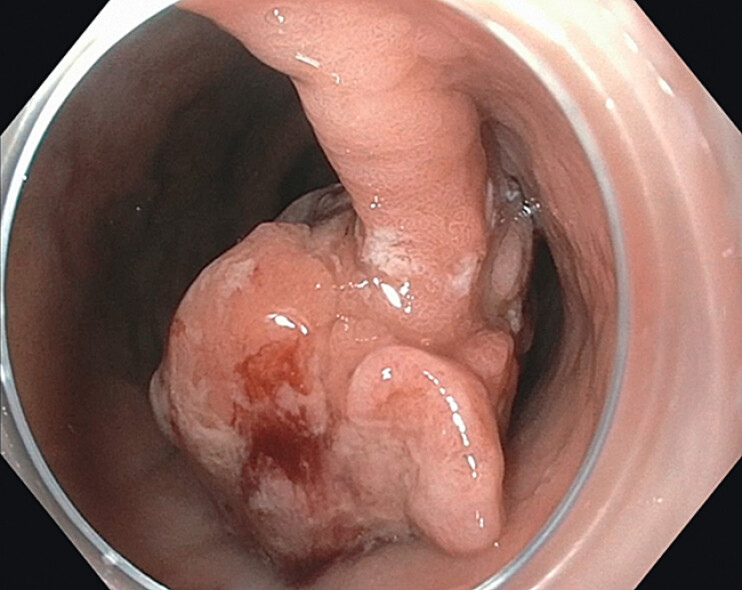
Endoscopic submucosal dissection (ESD) of a 40 mm gastric adenocarcinoma (Paris ISP, JNET 2B) on the lesser curvature of the proximal gastric body.

After several failed retrieval attempts using an endoscopic retrieval net and overtube, the intact specimen was removed via a double-channel gastroscope with dual forceps to elongate the lesion and a distal attachment foreign body retrieval hood affixed to the distal end of the gastroscope, facilitating passage through the LES. This approach minimized trauma, reduced the risk of spasm-induced fragmentation, and preserved specimen integrity. Pathology confirmed negative margins. Given the lack of standardized methods for retrieving large gastric lesions, this technique provides a practical solution for intact extraction in future ESD cases.

Endoscopy_UCTN_Code_TTT_1AO_2AN

## Notes

Endoscopy E-Videos https://eref.thieme.de/e-videos
*E-Videos* is an open access online section of the journal *Endoscopy* , reporting on interesting cases and new techniques in gastroenterological endoscopy. All papers include a high-quality video and are published with a Creative Commons CC-BY license. Endoscopy E-Videos qualify for HINARI discounts and waivers and eligibility is automatically checked during the submission process. We grant 100% waivers to articles whose corresponding authors are based in Group A countries and 50% waivers to those who are based in Group B countries as classified by Research4Life (see: https://www.research4life.org/access/eligibility/ ). This section has its own submission website at https://mc.manuscriptcentral.com/e-videos .

